# Landscape Simplification Modifies Trap-Nesting Bee and Wasp Communities in the Subtropics

**DOI:** 10.3390/insects11120853

**Published:** 2020-12-01

**Authors:** Rachele S. Wilson, Sara D. Leonhardt, Chris J. Burwell, Chris Fuller, Tobias J. Smith, Benjamin F. Kaluza, Helen M. Wallace

**Affiliations:** 1 Genecology Research Centre, University of the Sunshine Coast, 90 Sippy Downs Dr, Sippy Downs, QLD 4556, Australia; 2 Environmental Futures Research Institute, Griffith University, 170 Kessels Rd, Nathan, QLD 4111, Australia; chris.burwell@qm.qld.gov.au (C.J.B.); helen.wallace@griffth.edu.au (H.M.W.); 3 Department of Ecology and Ecosystem Management, Technical University of Munich, Hans-Carl-von-Carlowitz-Platz 2, 85354 Freising, Germany; leonhardt@wzw.tum.de; 4 Biodiversity Program, Queensland Museum, PO Box 3300, South Brisbane, QLD 4101, Australia; 5 School of Environment and Science, Griffith University, Nathan, QLD 4111, Australia; 6 Kin Kin Native Bees, Main St, Kin Kin, QLD 4571, Australia; clan03@bigpond.net.au; 7 School of Biological Sciences, University of Queensland, St Lucia, QLD 4072, Australia; tobias.smith@uqconnect.edu.au; 8 Department of Public Technology and Innovation Planning, Fraunhofer Institute for Technological Trend Analysis INT, Appelsgarten 2, 53879 Euskirchen, Germany; benjamin.kaluza@int.fraunhofer.de

**Keywords:** bee hotels, Hymenoptera, land use change, habitat loss, habitat complexity, bee decline, countryside

## Abstract

**Simple Summary:**

Many bees and wasps are important pollinators and natural pest controllers. Habitat loss is a major threat to bee and wasp conservation, but little is known about how this impacts tropical bees and wasps. This study aimed to determine how habitat loss affects solitary bees and wasps in tropical agricultural landscapes and how they change with the seasons. Solitary bees and wasps can be monitored using trap nests, popularly known as “bee hotels”. We installed bee hotels in forests and orchards and checked them every season over two years. We found 41 species of bees and wasps nesting in bee hotels. Importantly, five species of bees and 14 species of wasps were found only in forests, mostly species with particular food or nesting requirements. More species of bees and wasps used the hotels in the wet season (spring-summer). Our study suggests that solitary bees and wasps with special resource requirements are vulnerable to habitat loss in tropical agricultural landscapes.

**Abstract:**

(1) Background: Landscape simplification is a major threat to bee and wasp conservation in the tropics, but reliable, long-term population data are lacking. We investigated how community composition, diversity, and abundance of tropical solitary bees and wasps change with landscape simplification (plant diversity, plant richness, distance from forest, forest cover, and land use type) and season. (2) Methods: We installed 336 timber and cob trap nests in four complex forests and three simplified orchards within the subtropical biodiversity hotspot of south-east Queensland, Australia. Trap nests were replaced every season for 23 months and all emergents identified. (3) Results: We identified 28 wasp species and 13 bee species from 2251 brood cells. Bee and wasp community composition changed with landscape simplification such that large, ground-nesting, and spider-hunting species were present in all landscapes, while those with specialist resource requirements and (clepto) parasitoids were present only in complex landscapes. Abundance and diversity of bees and wasps were unaffected by landscape simplification but increased with rainfall. (4) Conclusions: This study highlights the need for multi-year studies incorporating nuanced measures such as composition with a focus on functional diversity to detect changes bee and wasp populations.

## 1. Introduction

Simplified landscapes are landscapes with little variation in land cover types, vertical vegetation structure, and plant diversity [1,2]. Agricultural expansion is the largest contributor to landscape simplification worldwide [3,4]. Simplified landscapes provide less resource diversity, niches, and species diversity than complex landscapes such as forests [2,5,6]. Landscape simplification is known to alter the abundance and diversity of many taxonomic groups, including birds [7], mammals [8], and invertebrates such as bees and wasps [9]. Bees and wasps provide essential pollination and pest management services, yet can be very sensitive to landscape simplification [10,11]. Maintaining bee and wasp communities in simplified landscapes, such as agroecosystems, therefore requires an understanding of their responses to respective changes in the landscape.

Natural or non-crop land in agriculture (e.g., forest, riparian vegetation, and fallow fields) acts as reservoirs for biodiversity and provides resources for bees and wasps [10,12,13,14]. Wild bee abundance and richness is lower in farms with little natural land use, resulting in lower flower visitation and crop yields [13,14]. Similarly, wasp diversity, abundance, and activity in fields can decrease with increasing distance to, or between patches of natural vegetation [10,12]. However, not all bee and wasp taxa are affected by landscape simplification in the same way, with traits such as nesting strategy (above- or below-ground) or sociality largely determining responses [15,16,17,18,19]. Furthermore, most research on bee and wasp responses to landscape simplification has been conducted in temperate regions of North America, Europe, and South America (see meta-analyses by [11,20,21,22]). Similar studies of bee and wasp responses in tropical and subtropical agroecosystems are rare [21], despite much of the tropics undergoing significant land use change [3].

Tropical regions have diverse bee and wasp faunas with potentially many thousands of species still waiting to be described [23,24]. Landscape simplification is considered a major threat to bee and wasp conservation in the tropics, but reliable population data is still needed to support further conclusions [23]. Nevertheless, some research suggests that different aspects of forests play an important role in tropical bee and wasp communities. For example, bee and wasp diversity and composition differs between forest and agricultural land uses in Brazil [25], Ecuador [26], and Costa Rica [15]. Within forests, bee and wasp diversity has been found to increase with canopy cover and fragment size in Australia [17,27], Colombia [28], and Mexico [29]. However, the longest tropical studies (two years), both in Brazil, show conflicting results with landscape simplification such as both increased [6] and decreased bee abundance [16]. This highlights the need for more long-term studies in tropical regions to understand the effects of landscape simplification.

Studies of bee or wasp responses to landscape simplification have largely been limited in their temporal observations, particularly in agricultural landscapes where sampling typically occurs over only one season of crop flowering (e.g., [30,31]). Observations across multiple seasons are important for showing changes in communities and thus ecosystem functions, such as from pollination by bee-dominated communities in spring to predation by wasp-dominated communities in summer [32]. The increasing use of trap nests for research presents opportunities to efficiently lengthen observations to several seasons or years (e.g., [6,33]), thus enabling more detailed, long-term investigations into solitary bee and wasp communities. Trap nests (also called “bee hotels” or “Fabre’s hives”) are artificially constructed nesting boxes for the study and management of solitary bees, wasps, and other insects (see reviews by [21,34]). Most trap-nest studies have measured responses of above-ground nesting taxa (i.e., those that nest in reeds or timber cavities), despite “below-ground nesters” dominating solitary bee species [24]. The few studies that have included below-ground nesters have used soil squares in situ [35] or portable cob structures as trap nests [36].

This study aimed to determine how landscape simplification and seasonal variability influence trap-nesting bee and wasp communities in the subtropics, using the biodiversity hotspot of south-east Queensland [37], Australia, as a case study. We asked how does community composition, diversity, and abundance of above- and below-ground nesting bees and wasps change with landscape simplification and season? As bee and wasp diversity can increase with landscape complexity in other bioregions, we hypothesise that: (1) community composition of bees and wasps will change with landscape simplification; (2) bee and wasp species diversity will be reduced in simplified landscapes; (3) bee and wasp abundance will be reduced in simplified landscapes; and (4) abundance and diversity of bees and wasps will vary with the seasons, decreasing in the cool, dry months.

## 2. Materials and Methods

**Figure 1:**
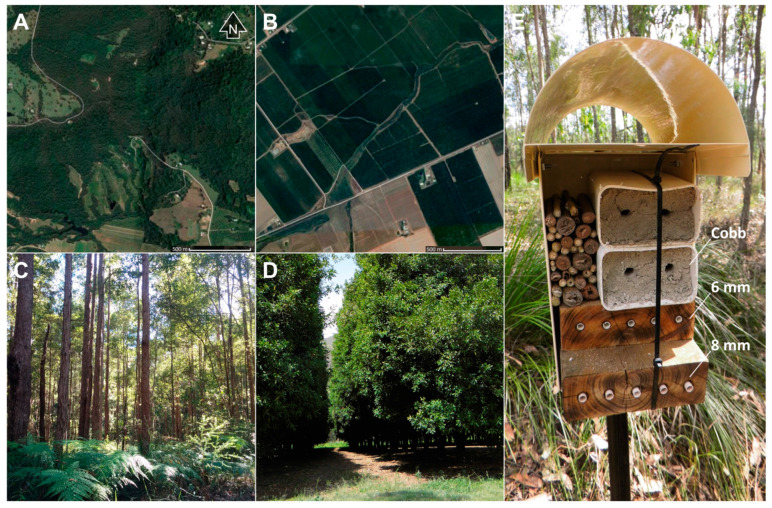
Study landscapes and trap nests. View of a forest (**A**) and orchard (**B**) landscape used in this study; habitat structure within a forest (**C**) and orchard (**D**) site; and an example of the trap nest units deployed in all sites (**E**) with cob blocks, pithy stems, short timber (100 mm long × 6 mm diameter) and long timber (150 mm long × 8 mm diameter) trap nests.

Seven study sites were established in south-east Queensland, Australia (−24°38′ to −27°29′ S, 152°6′ to 153°6′ E). Sites consisted of natural forests dominated by an overstorey of *Eucalyptus* and *Corymbia* species (four sites) or orchards that consisted of a complex matrix of commercial macadamia plantings (*Macadamia integrifolia* Maiden and Betche × *M. tetraphylla* Johnson) and forest fragments (three sites, Figure 1). We measured landscape simplification with the following metrics: dominant land use, plant richness, percentage of land cover type (forest and orchard), and distance to forest [13,19] (Table 1). Study sites selected had ≥75% of target forest or orchard cover [38]. There was a minimum distance of 1 km between all sites, 6 km between sites of different land use types, and approximately 200 km between northern and southern blocks of sites. Plant species at study sites were described by Kaluza et al. [38,39]. Forest sites generally had twice the plant species richness of orchard sites (Table 1) and a more complex vertical structure (Figure 1).

Trap nests were installed in all sites in October 2016. Trap nests were housed in metal units, fixed to steel pickets, 1 m above the ground, with a minimum distance between each unit of 5 m. All trap nests were oriented in the same direction (north-east) to face away from the prevailing winds of the cooler months and to reduce irregular colonization of cavities [21]. Four units were installed at each site, with each unit comprising 12 possible nests (10 timber cavities and 2 cob blocks). In total, 336 trap nests (16 units × 12 trap nests across the four forest sites and 12 units × 12 trap nests across the three orchard sites) were available for occupation across all sites in the first season, followed by 264 trap nests (12 units × 12 trap nests across forest sites and 10 units × 12 trap nests across orchard sites) per season, due to the loss of six units in February 2017 to theft and damage.

Three different nesting substrates were provided in each unit to accommodate nesting preferences of “above-ground” and “below-ground” nesters [18]. Pre-drilled timber blocks were provided for species that nest above-ground in existing hollow cavities (e.g., *Megachile* and *Hylaeus* spp.). This design was chosen to allow whole nest management, as the holes can be lined with paper tubes (“PaperTunnel” liners, Pollinator Paradise, Parma, ID, USA) for easy removal of nests and reuse of blocks. Cavities of different dimensions were used to attract differently sized bee species. Five short cavities (100 mm long × 6 mm diameter) and five long cavities (150 mm long × 8 mm diameter) drilled into timber blocks were provided for each nesting unit, with the entrances slightly charred before inserting tubes. Cob blocks were provided for below-ground nesting bees that prefer sandy loam soil types such as *Amegilla* spp. [40]. Two cob blocks (a 4:1 mixture of sand and clay) were provided for each nesting unit, with two starter entrance holes pre-formed in each block. Each cob block represents one possible nest, however, as there was no barrier within the cob to separate potential ground-nester tunnels. Mixed bunches of *Xanthorrhoea* sp. and *Lantana* sp. stems with soft pith were also included as substrates for species that excavate their own above-ground cavities (e.g., allodapine bees). These pithy stems showed no signs of occupation, however, and so were excluded from analyses (as in [41]).

Trap nest units were checked for occupation every three to four months from February 2017 to December 2018. Occupied trap nests were removed and replaced with fresh paper tubes or cob blocks each sampling period. Occupied nests were placed in mesh (cob) or organza (paper tubes) bags for transport until dissection. All paper tubes recovered were carefully dissected to record the number of brood cells (abundance, above-ground nesters), larvae, pupae, adults, nest material, provisions, and pests or parasites. We assumed that the number of nests gives an estimation of the number of nesting females and that the number of brood cells per nest is an estimation of fecundity [42], although it is possible that females constructed multiple nests per block and additional nests in other units or locations.

Dissected nests with larvae or pupae were resealed, transferred to clean organza bags (one nest per bag), and stored together in emergence boxes outdoors [43]. All cob nests recovered were stored outdoors in larger mesh bags (one block per bag) without dissection, to avoid destroying the fragile structures that support emergence in these bees. All emergents from cob nests were counted (abundance of below-ground nesters). All nests were checked monthly for emergents, which were then frozen at −18˚C and pinned for identification. Emergent bees and wasps were identified to species or morphospecies by CJB using available taxonomic literature and reference to collections in the Queensland Museum. Nests without adult emergents (e.g., dead larvae or pupae) were determined to likely host family or subfamily from nest materials or provisions. For example, nests with pollen and “cellophane” to Hylaeinae and of resin or leaves to Megachilidae. Nests that were vacated prior to field sampling were counted as “occupied” without further species determination or abundance record [44].

We tested the effects of seasonal variation (season, temperature, and precipitation) and landscape simplification (plant richness, distance from forest, forest cover, and land use type) on the response variables of bee and wasp species composition, diversity (richness and Shannon), and abundance using Generalized Linear Mixed Models (GLMMs). Daily maximum temperature and rainfall observations were retrieved from the Australian Bureau of Meteorology (bom.gov.au/climate/data/index.shtml) and averaged for weather stations closest to the northern (Station No. 039128, 14 km south of P3) and southern (Station No. 040861, 15 km north of F3) sites over each sampling period. Season was defined as the season in which trap nests were sampled: summer (December-February), autumn (March-May), winter (June-August), and spring (September-November). Bee and wasp species diversity was calculated using Shannon’s diversity index: $H=\sum_{i=1}^{s}-\left(P_{i}\times \;\ln P_{i}\right)$, where *s* is the total number of species and $P_{i}$ is the number of individuals of “species i” per site, divided by the total number of individuals for all species (Data S1). Bee abundance was segregated according to nesting strategy (above-ground and below-ground nesters), to account for different approaches used to measure abundance (i.e., number of brood cells vs. number of emergents) (Data S1). Bee diversity and richness was aggregated for all bees, as species identification was independent of nesting strategy (Data S1).

All analyses were performed in the statistical software ‘R’ (v 3.5.2, [45]) (Script S1). We used the vegan package to ordinate bee and wasp communities with non-metric multidimensional scaling (NMDS) using the *metaMDS*() function and visualised the data using Bray–Curtis distances with three dimensions and up to 999 permutations [46]. Effects of environmental variables on communities were tested individually with permutational multivariate ANOVAs using the *vegdist*() and *adonis*() functions, also with Bray-Curtis distance matrices [46,47]. Significant environmental variables were then fitted to NMDS models and visualised together with the *envfit*() function [46]. Similarity percentages to discriminate species between categorical groups was done using Bray-Curtis dissimilarities with the *simper*() function [46]. Data used for ordination and to construct GLMMs was analysed per trap nest unit. GLMMs were composed for each response variable, starting with the most complex model including all explanatory variables of interest and interactions between them, except where such variables were significantly correlated (i.e., season and precipitation; land cover, land use, and distance from forest). Where variables were significantly correlated, several models were composed each including only one of the correlated variables (e.g., model 1 = y ~ season*plant richness, model 2 = y ~ precipitation*plant richness). Random effects included in all models were trap nest unit, nested within site. An observation-level random effect was also added to GLMMs that were overdispersed [48]. Models were then simplified by consecutively dropping variables and comparing models with and without variables with likelihood ratio tests (χ^2^) using the *anova*() function until the most parsimonious model was reached. Final explanatory models were compared against null models (i.e., random factors only) to assess the significance levels of remaining variables [49]. Explanatory power of final models was determined from the variance of fixed (marginal *R*²) and random effects (conditional *R*²) with the MuMIn package [50]. Differences between levels of categorical explanatory factors were compared with Tukey’s post hoc tests using the *glht*() function [51].

## 3. Results

A total of 654 nests were constructed in trap nest cavities or cob blocks over the entire sampling period of 23 months (36% occupation of available cavities and cob). We recovered 590 of these nests for analysis. Nests that were incomplete (less than one cell) were excluded from further analyses (n = 28). Of the remaining 562 nests recovered (Table 2): 200 had at least one adult emergent from which we could identify the host species; 79 were determined to the most likely host (sub)family from nest materials and provisions; and 283 nests, all constructed with mud and/or provisioned with spiders, could not be determined beyond “wasps”.

### 3.1. Community Composition

Adult emergents from recovered nests were classified into 41 different species or morphospecies (Table S1). Trap nest communities were mostly comprised of wasps (28 species, 74% of nests) rather than bees (13 species, 26% of nests). Five bee species occurred only in forests and two bee species occurred only in orchards (Table 2). The remaining six bee species were recorded in both land uses (Table 2). Similarly, for wasps, seven species were observed in both land uses while 14 species were found only in forests and seven species only in orchards (Table 2).

Trap-nesting bee and wasp communities found at our sites exhibited a range of different functional traits. Most were nesting above-ground, including seven bee species (out of 13) and all wasps (Table 2). Bees were also found to use different nesting materials, including “cellophane” secretions (three hylaeine species), leaves (*Megachile simplex*), resin (three *Megachile* spp.), and the supplied cob mixture (ground-nesting Apidae and *Pachyprosopis* spp.) (Table 2). Non-parasitic wasp species provisioned brood cells with either spiders (13 species, Pompilidae and Crabronidae) or caterpillars (five Eumeninae species) and used mud or cob to construct nests (Table 2).

Most nests were provisioned by one host species, except for 25 nests which were either usurped or co-inhabited by two or more species. In particular, adults of the ground-nesting *Amegilla adelaidae* and both *Pachyprosopis* spp. often emerged from the same nest. Others were cavities filled with both wasp (*Pison* spp.) and bee (*Megachile mystacaena* or *Hylaeus* spp.) brood cells, usually in succession. Almost one-third of wasp species identified were parasitic, with most parasitising other wasp nests, except *Gasteruption* spp., which were found in nests of resin bees (*M. mystaceana*) and cellophane bees (Hylaeinae spp.). Other pests of bees included cleptoparasitic *Thyreus* spp., which were recorded in nests of both *Amegilla* species.

**Figure 2:**
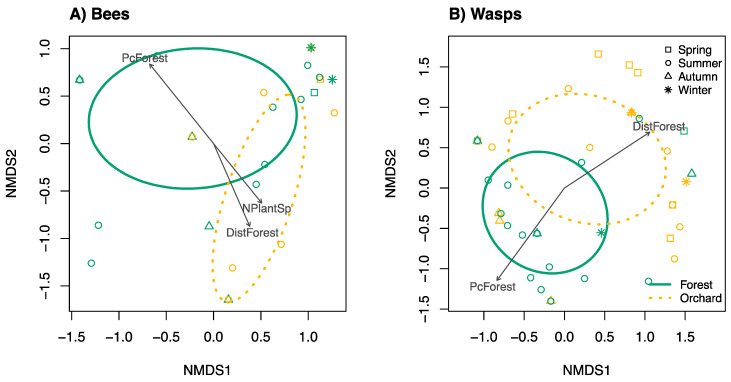
Changes of bee (**A**) and wasp (**B**) communities with landscape simplification and seasonal variation. Ellipses and points show forest (green) and orchard (orange) communities (standard deviation of point scores for groups of land use). Each point represents one trap nest unit in one season in one year (NMDS, Bray distance, 999 permutations). Vectors show direction of selected significant environmental factors. Bee community composition (**A**) differed with land use, but also distance to forest, forest cover, and plant richness. Composition of wasp communities (**B**) similarly differed with land use, percentage of forest cover, and distance to forest, but not plant richness.

Communities of bees and wasps were influenced differently by landscape simplification and season (Table 3). Bee communities were influenced by all environmental factors except rainfall, with the strongest effects being season, temperature, and amount of forest cover (Table 3). Bee community composition differed with land use, but also distance to forest, forest cover, and plant richness (Figure 2A). Wasp communities were influenced by all environmental factors except plant richness (Table 3). Composition of wasp communities differed with land use, percentage of forest cover, and distance to forest (Figure 2B).

A SIMPER analysis identified three bee species, *M. simplex*, *A. adelaidae,* and *M. mystacaena*, that collectively accounted for over 60% of the total bee community dissimilarity between forests and orchards (Table 4). These species were the most abundant individuals in trap nests, collectively totalling 203 of the 574 brood cells recovered from bee nests (Table 2). While *M. simplex* increased in abundance from forests to orchards, *A. adelaidae* was the opposite and *M. mystaceaena* was found only in forests. Similarly, four wasp species contributed the most to total wasp community dissimilarity between land uses: *Pison* sp. B, *Pison* sp. E, *Fabriogenia* sp. B and *Pison* sp. D (Table 4). Of these, *Fabriogenia* sp. B and *Pison* sp. B decreased in abundance from forests to orchards, *Pison* sp. E was found only in forests and *Pison* sp. D was found only in orchards (Table 2).

### 3.2. Species Diversity and Abundance

Variation in the species diversity and richness of bees and wasps was mostly explained by season or random factors rather than landscape simplification (Table 5). Bee diversity varied with season, and was higher in spring compared to autumn (Table 5). Bee species richness was not explained by any of our factors of interest, with similar numbers of species occurring across seasons and landscapes.

Bee abundance was unaffected by our measures of landscape simplification. The most abundant bee families were Megachilidae (49% of bee nests) and Colletidae (26%) (Table 2). Above-ground bee abundance increased with rainfall and varied with season, such that abundance was higher in summer compared to autumn (Table 5). Below-ground bee abundance similarly increased with rainfall and varied with season, with the lowest abundance in autumn (Table 5).

Wasp diversity was similarly influenced by season, being higher in summer compared to autumn (Table 5). Wasp species richness and abundance were best explained by random factors, with similar numbers of species and individuals occurring across seasons and landscapes. The most abundant wasp families were Crabronidae (57% of identified wasp nests) and Pompilidae (17%) (Table 2).

## 4. Discussion

We found that the composition of bee communities changed with land use type, amount of forest cover, distance to forest, plant diversity, and richness. Similarly, wasp community composition differed with land use type, amount of forest cover, and distance to forest, but not plant diversity or richness. The latter suggests that bees and wasps are affected differently by landscape simplification and require different resources to persist in subtropical agroecosystems. Importantly, five species of bees and 14 species of wasps were found only in forests, mostly species with specialist food or nesting requirements. In contrast to our expectations, we found diversity and abundance of bees and wasps varied with the seasons or rainfall, but not landscape simplification. Our study shows that, while overall numbers and diversity of tropical solitary bees and wasps may remain stable with landscape simplification, the composition of those communities is vulnerable to change.

The observed changes in bee and wasp community composition with landscape simplification was consistent with our first hypothesis. Similar changes were found by other trap-nest studies in tropical agroecosystems [6,15,25]. For example, plant diversity or richness was also a key determinant of bee community composition in Brazil [6]. The strong effect of plant diversity is probably due to changes in the availability of plant-based food (e.g., pollen) and nesting resources. Changes in wasp community composition with different forest metrics may indicate that there are forest-dependent species in each land use, including some species that favour less forest cover or shade (e.g., those found in the orchards only). Forests are important sources of food and nesting materials for wasps [21,52], where prey items may be more abundant irrespective of plant diversity and composition (e.g., caterpillars [53] and spiders [54]).

The composition in each land use is likely dependent on the traits of bee and wasp assemblages in our study system such as body size, foraging distance, resource use, and life history strategies such as parasitism (e.g., [16,28,55,56,57,58]). For example, the three bee and four wasp species that accounted for most dissimilarity between land uses (*A. adelaidae*, *M. mystacaena*, *M. simplex*, *Fabriogenia* sp. B, *Pison* sp. B, *Pison* sp. D, and *Pison* sp. E) were all large species, ground-nesters or spider-hunters. Those taxa present only in complex landscapes were mostly species with (known) specialist resource requirements (e.g., *M. mystacaena*, which needs resin-bearing trees) and the (clepto) parasitoids, which are dependent on the abundance and richness of their hosts [56,57]. Specialisation determines the breadth of resources exploited: specialists collect from a narrow range of (often related) resources, while generalists have a broader diet [59]. Specialists should be more vulnerable to decline when faced with shortages in their hosts, however, some can broaden their resource use during times of scarcity–if similar (morphologically or chemically) resources are available [60,61]. More comparative studies of bee and wasp resource use in different environments are needed to better understand the responses of specialists and generalists to changes in resource availability [62].

Contrary to our second and third hypotheses, the diversity and abundance of bees and wasps were not reduced in simplified landscapes. Instead, we saw similar abundances and numbers of bee and wasp species across sites with different levels of landscape complexity. Most studies found wild bee diversity and abundance is reduced in simplified agricultural landscapes (see reviews by [11,63]); or that diversity is reduced, but abundance is increased (e.g., with mass-flowering crops [64]). Our results may differ from these studies because of the inherently high biodiversity in the study region, even with landscape simplification. Of all our floristically simplified study sites, those with the least plant diversity (site “P2”; Table 1) still had at least 39 to 50 flowering plant species within 500 m of trap nests in addition to the mass-flowering macadamia. This could indicate that even simplified landscapes support relatively high trap-nesting bee and wasp diversity in the tropics—particularly of generalist species—as simplified tropical landscapes can still be relatively rich in plant species and thus provide more diet options compared with simplified temperate landscapes [62,65,66]. Furthermore, the comparatively short-term nature of similar trap-nest studies in tropical agroecosystems (e.g., 6–12 months [15,25]) may not capture the true variation of bee diversity and abundance following landscape simplification. However, this phenomenon could be restricted to cavity- or trap-nesting bees and wasps, due to their ability to nest in vertical soil or man-made cavities (e.g., studies using nets [16]; but see [15]).

Our hypothesis that diversity and abundance of bees and wasps will vary with the seasons, decreasing in the cool, dry months, was upheld. We found both above- and below-ground bee abundance to be positively associated with rainfall, being lowest in the cool, dry months of autumn. Diversity of bees and wasps was similarly lowest in autumn and highest in spring for bees and summer for wasps. In the tropics, many solitary bee species can reproduce continuously throughout the year, but particularly in the warm, wet season (e.g., in Ecuador [26]). Such patterns are likely also related to changes in resource availability [32]. As such, our findings suggest that bee and wasp communities in the subtropics follow similar seasonal patterns to those in the tropics.

We identified 28 wasp species (74% of nests) and 13 bee species (26% of nests) in trap nests. This is higher than the number of cavity-nesting species found in similar agroecological studies in temperate bioregions (e.g., [10,67]) and similar to those in the neotropics (e.g., [6]). Such parallels support the use of trap nests as indicators of bee and wasp diversity in different bioregions. Seeing more wasp than bee occupation is a common finding among trap nest studies (e.g., [10,44,68]). This may be because wasps outcompete bees for these nesting structures [68] or because wasps outnumber bees in general (~115,000 wasp species versus ~20,000 bee species) [69,70]. We found both wasps and bees often provisioned the same timber cavity, in succession, which could indicate that nests were being usurped by either host [71]. However, we also found two ground-nesting bee species commonly emerged in high numbers from the same cob blocks. This suggests that interspecies cohabitation among nesting aggregations (or, more likely, their emerging offspring) may be higher than expected.

## 5. Conclusions

Our study shows that the composition of tropical solitary bee and wasp communities is vulnerable to change with landscape simplification. However, overall abundance and diversity of trap-nesting species may remain stable. Large, ground-nesting bee and spider-hunting wasp species were present in all landscapes, while those with specialist resource requirements and most (clepto) parasitoids were present only in complex landscapes. Most trap-nest studies in tropical agroecosystems are comparatively short-term and may not capture effects of landscape simplification on bee and wasp communities. This study highlights the need for multi-year studies incorporating nuanced measures such as composition with a focus on traits to detect changes bee and wasp populations.

## Figures and Tables

**Table 1:** Landscape characteristics of study sites.

Land Use	Site	Plant Species Richness	Forest Cover (%)	Orchard Cover (%)	Distance to Forest (m)
Forest	F1	53	96.36	0	0
	F2	139	89.79	0	0
	F3	155	97.52	0	0
	F4	173	75.06	20.65	0
Orchard	P2	51	0	92.04	640
	P3	134	3.53	93.21	212
	P4	69	16.74	80.08	177

**Table 2:** Taxa identified in trap nests and their abundance in forests (“F”) and orchards (“O”). Some trap-nesting bees and wasps were found only in forests or orchards. Symbology shows aboveground nesting ^a^, belowground nesting ^b^, cleptoparasitic ^c.p.^, parasitic ^p^, cellophane ^c^, leafcutter ^l^ and resin-collecting ^r^ species. Abundance indicates total and mean (+/− se) number of adult emergents for ground-nesting bees and of brood cells for cavity-nesting bees and wasps. All specimens are determined to species and labelled by taxa names or letters “A” to “J” after “sp.” for unnamed taxa.

Family (% of Nests)	Species	Abundance		Number of Nests		
		Total Brood	Per Nest Mean ± SE	F	O	Total
*Bees*						
Apidae (25%)	*Amegilla (Zonamegilla) adelaidae* ^b^	85	5.67 (1.28)	9	6	15
	*Amegilla (Zonamegilla)* sp. B ^b^	4	2.00 (1.00)	1	1	2
	*Thyreus* cf. *caeruleopunctatus* ^b,c.p.^	1	1.00 (NA)	0	1	1
	*Thyreus nitidulus* ^b,c.p.^	2	1.00 (0.00)	2	0	2
Colletidae (26%)	*Hylaeus (Euprosopoides) ruficeps ruficeps* ^a,c^	1	1.00 (NA)	1	0	1
	*Hylaeus (Hylaeorhiza) nubilosus* ^a,c^	37	6.17 (2.02)	4	2	6
	*Hyleoides concinna* ^a,c^	13	4.33 (1.77)	3	0	3
	*Pachyprosopis (Parapachyprosopis) angophorae* ^b^	8	2.67 (0.88)	0	3	3
	*Pachyprosopis (Parapachyprosopis) indicans* ^b^	60	7.50 (2.91)	7	1	8
Megachilidae (49%)	*Megachile (Callomegachile) mystacaena* ^a,r^	69	3.00 (0.50)	23	0	23
	*Megachile (Eutricharaea) simplex* ^a,l^	49	4.45 (0.87)	2	9	11
	*Megachile (Rhodomegachile) deanii* ^a,r^	12	4.00 (0.00)	1	2	3
	*Megachile mackayensis* ^a,r^	3	1.50 (0.50)	2	0	2
*Wasps*						
Chrysididae (3%)	*Primeuchroeus* sp. ^a,p^	4	1.33 (0.33)	0	3	3
Crabronidae (57%)	*Pison* sp. A ^a^	25	5.00 (1.31)	1	4	5
	*Pison* sp. B ^a^	92	3.54 (0.30)	13	13	26
	*Pison* sp. C ^a^	9	2.25 (0.63)	1	3	4
	*Pison* sp. D ^a^	77	7.70 (0.60)	0	10	10
	*Pison* sp. E ^a^	93	5.81 (0.68)	16	0	16
	*Pison* sp. F ^a^	11	3.67 (0.88)	0	3	3
	*Pison* sp. G ^a^	1	1.00 (NA)	1	0	1
	*Pison* sp. H ^a^	1	1.00 (NA)	1	0	1
	*Pison* sp. I ^a^	1	1.00 (NA)	0	1	1
	*Pison* sp. J ^a^	5	5.00 (NA)	1	0	1
Gasteruptiidae (4%)	*Gasteruption* sp. A ^a,p^	4	1.00 (0.00)	4	0	4
	*Gasteruption* sp. B ^a,p^	1	1.00 (NA)	1	0	1
Mutillidae (4%)	Mutillidae sp. A ^a,p^	2	1.00 (0.00)	1	1	2
	Mutillidae sp. B ^a,p^	1	1.00 (NA)	1	0	1
	Mutillidae sp. C ^a,p^	1	1.00 (NA)	1	0	1
	Mutillidae sp. D ^a,p^	1	1.00 (NA)	1	0	1
Perilampidae (2%)	*Perilampus* sp. ^a,p^	2	1.00 (0.00)	1	1	2
Pompilidae (17%)	*Fabriogenia* sp. A ^a^	7	2.33 (1.33)	1	2	3
	*Fabriogenia* sp. B ^a^	64	4.57 (0.66)	10	4	14
	*Fabriogenia* sp. C ^a^	8	4.00 (3.00)	2	0	2
	*Irenangelus* sp. ^a,c.p.^	1	1.00 (NA)	1	0	1
Sphecidae (3%)	*Isodontia* sp. ^a^	3	1.00 (0.00)	0	3	3
Vespidae (12%)	*Anterhynchium (Epiodynerus) nigrocinctus* ^a^	31	5.17 (0.40)	0	6	6
	*Anterhynchium (Epiodynerus) tamarinum* ^a^	4	1.33 (0.33)	0	3	3
	Eumeninae sp. B ^a^	1	1.00 (NA)	1	0	1
	Eumeninae sp. D ^a^	16	8.00 (5.00)	2	0	2
	*Paralastor* sp. ^a^	4	2.00 (1.00)	2	0	2
Sub total		814		118	82	200
Bee nests identified to (sub)family		230		53	15	68
Wasp nests identified to (sub)family		48		8	3	11
Unidentified wasp nests		1159		170	113	283
Total		2251		349	213	562

**Table 3:** Environmental effects on composition of bee and wasp communities. Bee communities were influenced by all environmental factors except rainfall. Wasp communities were influenced by all environmental factors except plant richness. Parameters reported are from permutational mANOVAs and non-metrical multidimensional scaling (NMDS) based on Bray–Curtis similarity distances between abundance of each species per trap nest unit, per season (999 permutations, 3 dimensions).

Community	Stress	Explanatory Variables	*F*	*R* ^2^	*p*
Bees	0.14	Season	1.809	0.14	0.009
		Temperature	4.352	0.11	0.001
		Rainfall	1.544	0.04	0.121
		Land use	3.135	0.08	0.003
		Forest cover	3.804	0.1	0.001
		Distance to forest	3.051	0.08	0.001
		Plant richness	3.328	0.09	0.002
Wasps	0.2	Season	1.787	0.1	0.004
		Temperature	2.041	0.04	0.017
		Rainfall	2.408	0.04	0.007
		Land use	2.727	0.05	0.002
		Forest cover	2.779	0.05	0.002
		Distance to forest	2.826	0.05	0.004
		Plant richness	0.994	0.02	0.439

**Table 4:** Contributions of bee and wasp species to forest and orchard communities. Proportions represent each species’ contribution to the total community dissimilarity between forests and orchards. Most of the dissimilarity between land uses is due to just three bee species (62%, cumulative): *M. simplex*, *A. adelaidae,* and *M. mystacaena* (found only in forests). For wasps, most dissimilarity is contributed by four wasp species (60%): *Pison* sp. B, *Pison* sp. E (found only in forests), *Fabriogenia* sp. B and *Pison* sp. D (found only in orchards).

Species	Proportion	
	Mean	Cumulative
Bees		
*Megachile (Eutricharaea) simplex*	0.2356	0.2515
*Amegilla (Zonamegilla) adelaidae*	0.1966	0.4613
*Megachile (Callomegachile) mystacaena*	0.1522	0.6237
*Pachyprosopis (Parapachyprosopis) indicans*	0.1032	0.7339
*Hylaeus (Hylaeorhiza) nubilosus*	0.0938	0.8340
*Megachile (Rhodomegachile) deanii*	0.0658	0.9042
*Pachyprosopis (Parapachyprosopis) angophorae*	0.0298	0.9361
*Hyleoides concinna*	0.0226	0.9602
*Amegilla (Zonamegilla)* sp. B	0.0164	0.9778
*Hylaeus (Euprosopoides) ruficeps ruficeps*	0.0064	0.9846
*Megachile mackayensis*	0.0052	0.9902
*Thyreus* cf. *caeruleopunctatus*	0.0049	0.9955
*Thyreus nitidulus*	0.0042	1.0000
Wasps		
*Pison* sp. B	0.1895	0.2049
*Pison* sp. E	0.1562	0.3738
*Fabriogenia* sp. B	0.1253	0.5093
*Pison* sp. D	0.0917	0.6084
*Anterhynchium (Epiodynerus) nigrocinctus*	0.0677	0.6817
*Pison* sp. A	0.0560	0.7423
*Pison* sp. C	0.0328	0.7778
*Pison* sp. F	0.0298	0.8101
*Fabriogenia* sp. A	0.0259	0.8381
*Fabriogenia* sp. C	0.0195	0.8592
Eumeninae sp. D	0.0171	0.8778
*Isodontia* sp.	0.0166	0.8957
*Gasteruption* sp. A	0.0136	0.9105
*Paralastor* sp.	0.0110	0.9225
*Primeuchroeus* sp.	0.0105	0.9339
*Anterhynchium (Epiodynerus) tamarinum*	0.0105	0.9453
Mutillidae sp. A	0.0090	0.9551
*Pison* sp. J	0.0089	0.9647
*Irenangelus* sp.	0.0061	0.9714
*Gasteruption* sp. B	0.0048	0.9819
Eumeninae sp. B	0.0048	0.9766
*Perilampus* sp.	0.0036	0.9858
*Pison* sp. G	0.0028	0.9889
*Pison* sp. H	0.0028	0.9920
Mutillidae sp. B	0.0024	0.9947
Mutillidae sp. C	0.0016	0.9965
Mutillidae sp. D	0.0016	0.9983
*Pison* sp. I	0.0015	1.0000

**Table 5:** Species richness, diversity, and abundance of bees and wasps in response to landscape simplification and season. Explanatory power of most parsimonious GLMMs including statistically significant variables is shown as variance of fixed (marginal R²) and random effects (conditional R²). Significance levels of variables were assessed by likelihood-ratio tests (LRT) against null models (i.e., random factors only). Below-ground bee abundance is modelled as a presence-absence (binary) response. There was no significant model for bee richness, wasp richness, or wasp abundance.

Response	Explanatory	∆ R^2^		LRT			Tukey Post-hoc	
		Marginal	Conditional	X^2^	df	*p*	Levels (Direction)	*p*
Bee diversity	Season	0.15	0.40	8.018	3	0.0456	Spring > Autumn	0.0461
Above-ground bee abundance	Rainfall	0.19	0.99	10.289	1	0.0013	(+)	
	Season	0.19	0.98	15.016	3	0.0018	Summer > Autumn	0.0114
Below-ground bee abundance	Rainfall	0.16	0.16	5.062	1	0.0244	(+)	
	Season	0.96	0.99	43.801	3	<0.0001	Spring > Autumn	<0.0001
							Summer > Autumn	<0.0001
							Summer > Spring	<0.0001
							Winter > Spring	<0.0001
							Winter > Summer	<0.0001
							Winter > Autumn	<0.0001
Wasp diversity	Season	0.20	0.31	12.249	3	0.0065	Summer > Autumn	0.0033

## Supplementary Materials

The following are available online at https://www.mdpi.com/2075-4450/11/12/853/s1, Table S1: Taxonomy of bees and wasps identified in this study, Data S1: Spreadsheet of all data used in this study, Script S1: R scripts used to analyse and visualise Data S1.
