# Supplementary material for: Landscape Simplification Modifies Trap-Nesting Bee and Wasp Communities in the Subtropics
Source: Insects. 2020 Dec 1;11(12):853. doi: 10.3390/insects11120853 (PMC7760584; doi:10.3390/insects11120853)
Supplement: Supplementary file 1 [file insects-11-00853-s001.zip › insects-1008898-supplementary/insects-1008898-supple-conversion/Table S1.docx]

Table S1. Taxonomy of bees and wasps identified in this study.

| **Superfamily** | **Family** | **Subfamily** | **Species** |
| --- | --- | --- | --- |
| Apoidea | Apidae | Apinae | *Amegilla (Zonamegilla)* *adelaidae* |
| Apoidea | Apidae | Apinae | *Amegilla (Zonamegilla)* sp. B |
| Apoidea | Apidae | Apinae | *Thyreus* cf. *caeruleopunctatus* |
| Apoidea | Apidae | Apinae | *Thyreus nitidulus* |
| Apoidea | Colletidae | Euryglossinae | *Pachyprosopis (Parapachyprosopis)* *angophorae* |
| Apoidea | Colletidae | Euryglossinae | *Pachyprosopis* *(Parapachyprosopis)* *indicans* |
| Apoidea | Colletidae | Hylaeinae | *Hylaeus* *(Euprosopoides)* *ruficeps* |
| Apoidea | Colletidae | Hylaeinae | *Hylaeus (Hylaeorhiza) nubilosus* |
| Apoidea | Colletidae | Hylaeinae | *Hyleoides concinna* |
| Apoidea | Megachilidae | Megachilinae | *Megachile (Callomegachile)* *mystacaena* |
| Apoidea | Megachilidae | Megachilinae | *Megachile (Eutricharaea)* *simplex* |
| Apoidea | Megachilidae | Megachilinae | *Megachile* *(Rhodomegachile) deanii* |
| Apoidea | Megachilidae | Megachilinae | *Megachile mackayensis* |
| Apoidea | Crabronidae | Crabroninae | *Pison* sp. A |
| Apoidea | Crabronidae | Crabroninae | *Pison* sp. B |
| Apoidea | Crabronidae | Crabroninae | *Pison* sp. C |
| Apoidea | Crabronidae | Crabroninae | *Pison* sp. D |
| Apoidea | Crabronidae | Crabroninae | *Pison* sp. E |
| Apoidea | Crabronidae | Crabroninae | *Pison* sp. F |
| Apoidea | Crabronidae | Crabroninae | *Pison* sp. G |
| Apoidea | Crabronidae | Crabroninae | *Pison* sp. H |
| Apoidea | Crabronidae | Crabroninae | *Pison* sp. I |
| Apoidea | Crabronidae | Crabroninae | *Pison* sp. J |
| Apoidea | Sphecidae | Sphecinae | *Isodontia* sp. |
| Chalcidoidea | Chrysididae | Chrysidinae | *Primeuchroeus* sp. |
| Chalcidoidea | Perilampidae | Perilampinae | *Perilampus* sp. |
| Evanioidea | Gasteruptiidae | Gasteruptiinae | *Gasteruption* sp. A |
| Evanioidea | Gasteruptiidae | Gasteruptiinae | *Gasteruption* sp. B |
| Vespoidea | Mutillidae |  | Mutillidae sp. A |
| Vespoidea | Mutillidae |  | Mutillidae sp. B |
| Vespoidea | Mutillidae |  | Mutillidae sp. C |
| Vespoidea | Mutillidae |  | Mutillidae sp. D |
| Vespoidea | Pompilidae | Ceropalinae | *Irenangelus* sp. |
| Vespoidea | Pompilidae | Pepsinae | *Fabriogenia* sp. A |
| Vespoidea | Pompilidae | Pepsinae | *Fabriogenia* sp. B |
| Vespoidea | Pompilidae | Pepsinae | *Fabriogenia* sp. C |
| Vespoidea | Vespidae | Eumeninae | *Anterhynchium (Epiodynerus)* *nigrocinctus* |
| Vespoidea | Vespidae | Eumeninae | *Anterhynchium (Epiodynerus) tamarinum* |
| Vespoidea | Vespidae | Eumeninae | Eumeninae sp. B |
| Vespoidea | Vespidae | Eumeninae | Eumeninae sp. D |
| Vespoidea | Vespidae | Eumeninae | *Paralastor* sp. |
